# Generation of an MC3R knock-out pig by CRSPR/Cas9 combined with somatic cell nuclear transfer (SCNT) technology

**DOI:** 10.1186/s12944-019-1073-9

**Published:** 2019-05-28

**Authors:** Yajun Yin, Haiyang Hao, Xingbin Xu, Liangcai Shen, Wenjing Wu, Jin Zhang, Qiuyan Li

**Affiliations:** 1 College of Biological, Chemical Science and Engineering, Jiaxing University, Jiaxing, 314001 China; 2 State Key Laboratory of Agrobiotechnology & College of Biological Sciences, China Agricultural University, Beijing, 100193 China; 3 College of life science and biotechnology, Hebei Normal University of Science and Technology, Qinhuangdao, 066004 China

**Keywords:** MC3R, CRISPR/Cas9, Knockout, Pig

## Abstract

**Background:**

Melanocortin 3 receptor (MC3R), a rhodopsin-like G protein-coupled receptor, is an important regulator of metabolism. Although MC3R knock-out (KO) mice and rats were generated in earlier studies, the function of MC3R remains elusive. Since pig models have many advantages over rodents in metabolism research, we generated an MC3R-KO pig using a CRSPR/Cas9-based system combined with somatic cell nuclear transfer (SCNT) technology.

**Method:**

Four CRSPR/Cas9 target vectors were constructed and then their cleavage efficiency was tested in porcine fetal fibroblasts (PFFs). The pX330-sgRNA1 and pX330-sgRNA4 vectors were used to co-transfect PFFs to obtain positive colonies. PCR screening and sequencing were conducted to identify the genotype of the colonies. The biallelically modified colonies and wild-type control colonies were used simultaneously as donor cells for SCNT. A total of 1203 reconstructed embryos were transferred into 6 surrogates, of which one became pregnant. The genotypes of the resulting piglets were determined by PCR and sequencing, and off-target effects in the MC3R KO piglets were detected by sequencing. Then, offspring were obtained through breeding and six male KO pigs were used for the growth performance analysis.

**Results:**

Four vectors were constructed successfully, and their cleavage efficiencies were 27.96, 44.89, 32.72 and 38.86%, respectively. A total of 21 mutant colonies, including 11 MC3R^−/−^ and 10 MC3R^+/−^ clones, were obtained, corresponding to a gene targeting efficiency of 29.17%, with 15.28% biallelic mutations. A total of 6 piglets were born, and only two MC3R KO piglets were generated, one with malformations and a healthy one. No off-target effects were detected by sequencing in the healthy mutant. Six male MC3R KO pigs were obtained in the F2 generation and their body weight and body fat were both increased compared to wild-type full siblings.

**Conclusion:**

A MC3R KO pig strain was generated using the CRSIPR/Cas9-based system, which makes it possible to study the biological function of MC3R in a non-rodent model.

**Electronic supplementary material:**

The online version of this article (10.1186/s12944-019-1073-9) contains supplementary material, which is available to authorized users.

## Background

The melanocortin receptor (MCR) family belongs to the rhodopsin-like G protein-coupled receptor family with seven conserved domains, and plays a pivotal physiological role in energy homeostasis [1]. Melanocortin 3 and melanocortin 4 receptors (MC3R and MC4R) are two neural melanocortin receptors. MC3R is primarily expressed in the hypothalamus and can also be found in the placenta, gut, heart, kidney, and peritoneal macrophages [2–5]. MC3R plays an essential role in many physiological processes, including fat metabolism, energy homeostasis, and the immune response [6, 7]. Several studies showed that polymorphisms of MC3R may be related to an increased risk of tuberculosis in South African and Korean populations [8, 9]. Another study revealed that MC3R mutations are closely associated with obesity [10]. Twenty-seven MC3R mutations have been reported to date, and all the evidence supports the hypothesis that MC3R is important for human energy homeostasis [10–13]. Nevertheless, the pathogenic role of MC3R in human obesity remains controversial [10]. MC4R is also expressed in the hypothalamus and has been recognized as a regulator of feeding behavior and energy expenditure [14, 15]. To clarify the respective roles of MC3R and MC4R in energy homeostasis, researchers generated MC3R and MC4R knockout (KO) rodent models. MC3R KO mice exhibited greater fat mass with reduced fat-free mass, so that the total body weight was not notably increased, and showed greater feeding efficiency (the ratio of weight gain to energy intake) [12]. By contrast, MC3R KO rats displayed hypophagia and their body weight decreased [13]. MC4R KO mice and rats showed similar phenotypes, exhibiting hyperphagia and increased body weight [13]. Interestingly, MC3R and MC4R double KO mice and rats displayed even higher levels of adiposity, as well as more severe glucose intolerance and hyperglycemia than their MC4R KO counterparts, which indicated that the functions of the two melanocortin receptors are not mutually redundant [12, 13]. Taken together, the data indicate that MC3R is an important regulator of metabolism, but more evidence is needed to unveil its exact biological function.

Currently, pigs are extensively used as a large-animal model for biomedical research because of their greater similarities with humans in terms of anatomy, physiology, and immunology, compared to rodents [11, 16, 17]. The advantages of pig models are most obvious in metabolism-related studies. Genome editing technology, such as zinc-finger nucleases (ZNFs) [18], and transcription tctivator-like effector nucleases (TALENs) [19], made the routine generation of genetically modified pig models feasible, even if labor-intensive. Recently, the clustered regularly interspaced short palindromic repeats (CRISPR) associated protein system (CRISPR/Cas system) has become the predominant technology of genome editing, simplifying the process further. By delivering the Cas9 nuclease complexed with a synthetic guide RNA (gRNA) into a cell, the cell’s genome can be cut at a desired location, allowing existing genes to be removed and/or new ones to be inserted [20, 21].

In this study, MC3R KO piglets were successfully generated using the CRISPR/Cas9 system combined with somatic cell nuclear transfer (SCNT) technology, providing a valuable non-rodent model to elucidate the physiological function of MC3R in the future.

## Materials and methods

### Animals

The experimental procedures used in this study, including the feeding, transport, sampling and surgery of the subject pigs, were approved by the Jiaxing University Experimental Animal Welfare Ethics Committee. All surgical procedures were performed under anesthesia, and all efforts were made to minimize animal suffering.

### Construction of the Cas9 and sgRNA vectors

**Fig. 1:**
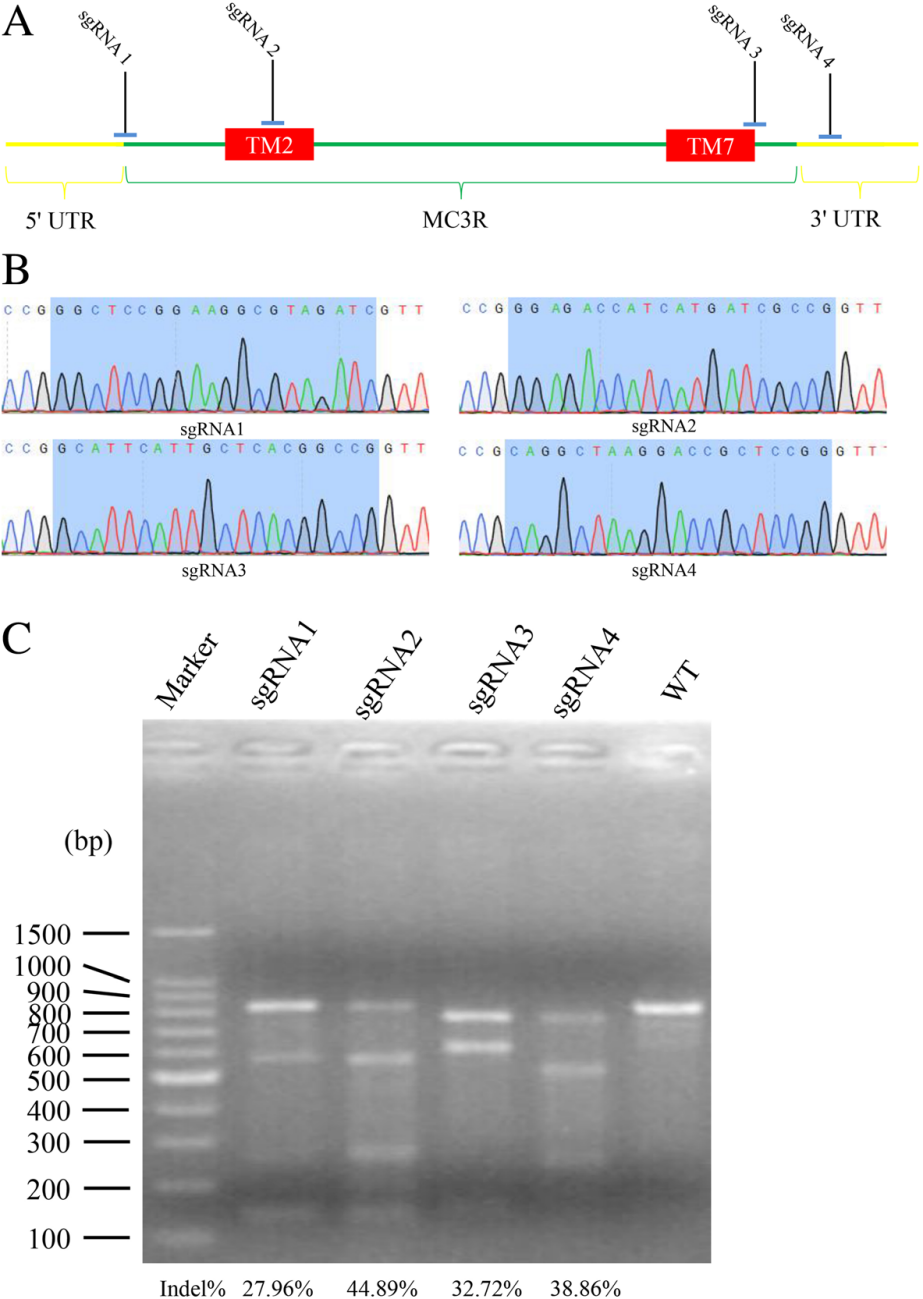
The selection of target sites and construction of targeting vectors. **a** The locations of the target sites in MC3R. sgRNA1 was located between the end of the 3’UTR and the beginning of MC3R, sgRNA2 was in the TM2, sgRNA3 contained part of TM7 and its surrounding sequences, sgRNA4 was in the 5’UTR. **b** Sequencing results of target sites in the CRISPR/Cas9 targeting vectors. Blue shaded areas indicate the target sequence. **c** PCR products and measurement of the cleavage efficiencies of the MC3R targeting vectors using the T7E1 assay

The sgRNA-Cas9 co-expressed vector pX330 (containing 2 *Bbs*I sites), pEGFP-N1 (including *eGFP*) and pL425 (containing a neomycin resistance gene) were obtained from Addgene. Four sgRNAs (sgRNA1, sgRNA2, sgRNA3 and sgRNA4) were selected in MC3R using CRISPR/Cas9 Design software (http://crispr.mit.edu/), targeting the CDS, 3′ UTR and 5′ UTR of MC3R in the pig genome (Fig. 1a and Table 1).

**Table 1:** The sgRNA sequences used in this study

sgRNA	Sequence (5′ → 3′)
sgRNA1	GCATTCATTGCTCACGGCCG GGG
sgRNA2	GGAGACCATCATGATCGCCG TGG
sgRNA3	GGCTCCGGAAGGCGTAGATC AGG
sgRNA4	CAGGCTAAGGACCGCTCCGG CGG

Note: PAM sequences are underlined

To construct the MC3R CRISPR/Cas9 target vector, the oligonucleotides sgRNA1F and sgRNA1R (Additional file 1: Table S1) were synthesized and directly annealed to yield a double-stranded DNA, which was ligated into the CRISPR/Cas9 expression vector between two *Bbs*I sites, and the resulting vector pX330-sgRNA1 confirmed by sequencing. The vectors pX330-sgRNA2, pX330-sgRNA3 and pX330-sgRNA4 were constructed using the same strategy (Fig. 1b).

### Cell culture, transfection and selection of porcine fetal fibroblasts (PFFs)

Porcine fetal fibroblasts (PFFs) were isolated from 30-day-old fetuses of Chinese Experimental Mini (CEM) pigs. The heads, tails, limbs and viscera of the fetuses were removed aseptically, and the remaining tissue was cultured in high glucose DMEM (Gibco, USA) with 15% FBS (Gibco, USA) and 1 × Penn/Strep solution (Gibco, USA) using previously described methods. Isolated PFFs were cultured in 10-cm dishes for 12 h and frozen in cell freezing medium (Gibco, USA). A day before transfection, PFFs were thawed and cultured to sub-confluency. Aliquots comprising 1 μg of the *MC3R* targeting vector and 1 μg of the pEGFP-N1 vector were used to co-transfect the PFFs by electroporation. After 24 h of recovery, the green fluorescence of the cells was monitored under a fluorescence microscope, after which the genomic DNA was extracted for PCR. The primer sequences are listed in Additional file 1: Table S2. Subsequently, the purified PCR products were tested using the T7E1 (NEB, USA) cleavage assay [22]. Briefly, a total of 250 ng of the purified PCR product was mixed with NEB Buffer 2, denatured, and annealed to allow formation of heteroduplexes using the following conditions: 95 °C for 5 min, cooling from 95 °C to 85 °C at 2 °C/s, 85 °C to 25 °C at 0.1 °C/s, and 4 °C hold. After reannealing, the products were digested with 1 μL of T7E1 at 37 °C for 15 min and separated on a 2% agarose gel stained with ethidium bromide. Cleavage efficiencies were calculated according to the densitometric grayscale values of the DNA bands.

To identify the large-fragment deletion in the pig genome, MC3R targeting vectors (pX330-sgRNA1 and pX330-sgRNA4) and pL452 (expressing neomycin resistance) were introduced into PFFs, and the cells were seeded into 10-cm dishes. After 48 h, 800 μg/mL of G418 (Gibco, USA) was added to select single colonies for 8–12 days. One half of the single colonies was cultured in 24-well plates for PCR screening, and the other half was transferred into 12-well plates for SCNT. The PCR screening primers used to amplify the target region were as follows: Forward: 5′-ATCTCTCAAGGGGTGTCTCCCG-3′, reverse: 5′- TGTTCTCAGGTGACCGCA TGAC-3′. The PCR conditions were as follows: 94 °C for 2 min, followed by 30 cycles of 95 °C for 10 s, 60 °C for 30 s, and 72 °C for 5 min, and a final 72 °C for 10 min. The PCR products were sequenced (Sangon Biotech, China) after ligation into a pMD19-T vector (Takara, Japan) according to the manufacturer’s instructions. Ninety-six individual clones were picked and sequenced.

### Generation and identification of the MC3R knock-out pigs

The oocytes for SCNT were collected from ovaries purchased from a local slaughterhouse, cultured for 42–44 h in vitro, and enucleated as described elsewhere [23]. Briefly, a single targeted PFF cell was transferred into the perivitelline space of the enucleated oocyte, after which the donor cell and recipient cytoplast were fused and activated to form a reconstructed oocyte. Then, the reconstructed oocytes were cultured in embryo-development medium for 4 h at 38.5 °C. About 1203 blastocysts were transplanted into the uteruses of six surrogate sows. Pregnancy was confirmed by ultrasound 30 days after transplantation and monitored until the perinatal period. All piglets were born by normal delivery.

The genomic DNA of the F0 piglets was isolated from an ear biopsy, and the genomic region surrounding the target site was amplified by PCR. The PCR conditions were as follows: 95 °C for 5 min, followed by 30 cycles of 95 °C for 10 s, 60 °C for 30 s, and 72 °C for 40 s, and a final 72 °C for 7 min. The PCR products were sequenced (Sangon Biotech, China) after insertion into a pMD19-T vector.

### Assay for off-target mutations

**Fig. 2:**
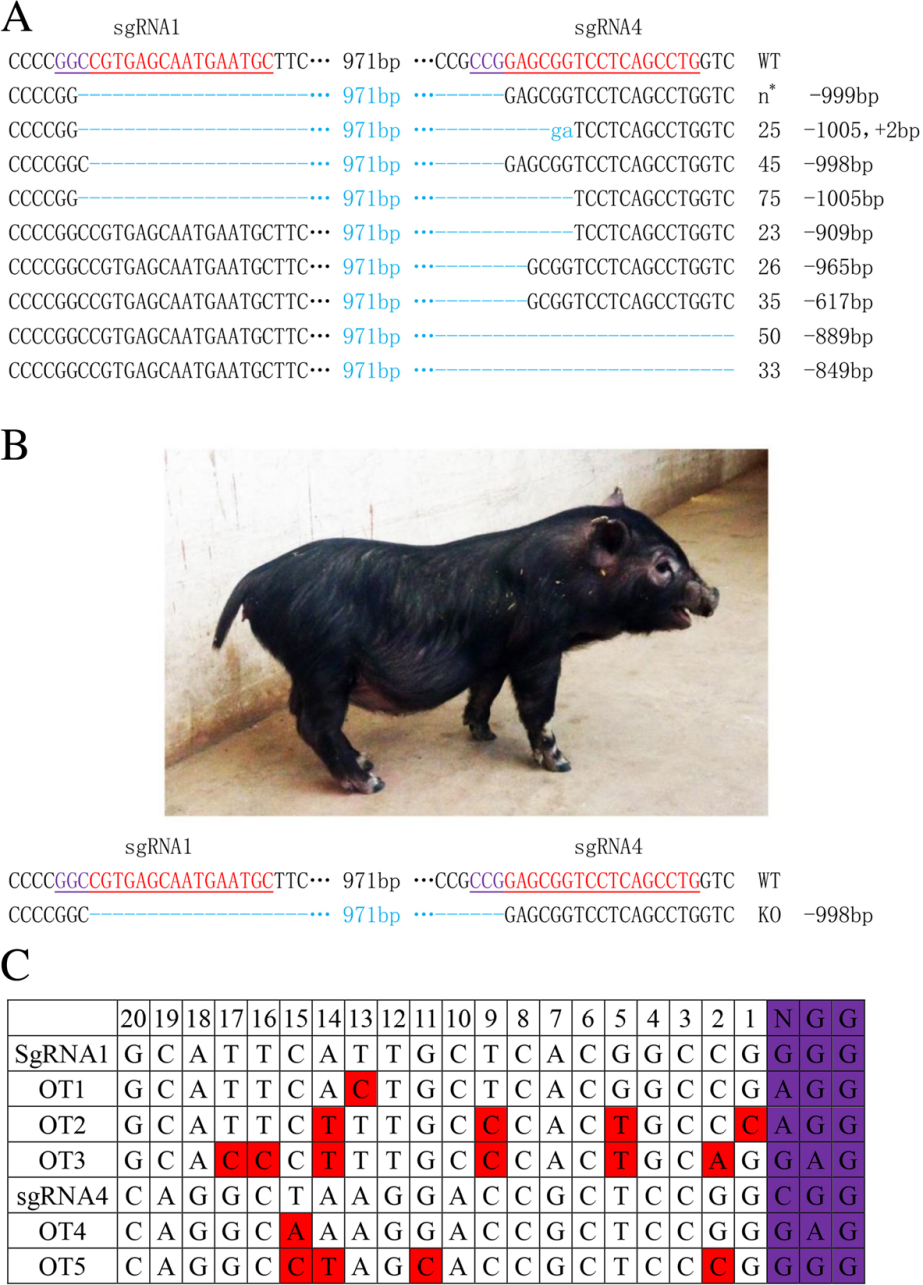
Identification of the MC3R KO pig. **a** Sequencing results of re-edited sgRNA1 and sgRNA4 in PFFs. “n^*^” stands for 9 samples (26, 46, 64, 65, 66, 69, 84, 93, and 94). **b** A photograph of the MC3R KO piglet (healthy, 50d), and sequence alignment of the deletion. **c** Sequencing results of potential off-target sites. There were no off-target mutations in the MC3R KO piglets

Potential off-target sites (OTSs) were predicted using CRISPR Design software, amplified form the genomic DNA of the MC3R KO piglet, and sequenced. Variants were identified by sequence alignment. Sequences of the potential OTSs are shown in Fig. 2c, and the primers used to amplify them are listed in Additional file 1: Table S3.

### Breeding of F2 offspring and growth performance analysis

The MC3R KO piglet was mated with wild-type sows at eight months old. Eighteen piglets were obtained from three mothers at the F1 generation. Then, five male pigs (MC3R^+/-^) were mated with five sows (MC3R^+/-^) to produce the F2 generation. Six males with MC3R^−/−^ genotype were selected for body weight and adiposity analysis, and compared with their full siblings with MC3R^+/+^ genotype.

## Results

### Construction of the targeting vector and test of its cleavage efficiency

Porcine fetal fibroblasts (PFFs) of Chinese Experimental Mini (CEM) pigs were used as donor cells for the generation of MC3R-knockout pigs. Since single nucleotide polymorphisms (SNPs) can potentially affect CRISPR/Cas9 efficiency, the genomic sequence of the MC3R locus in the CEM pig was analyzed by sequencing. Seven SNPs were found to differ from the MC3R sequence from GenBank (Additional file 1: Figure S1). Four sgRNAs were selected according to the MC3R sequence of the CEM pig using CRISPR Design software (Fig. 1a). The sgRNA1 encompassed the 5′ untranslated region (UTR) and first exon, the sgRNA2 was located in the transmembrane domain (TM) 2, the sgRNA3 encompassed TM7 and adjacent downstream sequence, and sgRNA4 was located in the 3′ UTR. Four pairs of oligonucleotides harboring each sgRNA and PAM sequence were synthesized and cloned into the pX330 vector (Additional file 1: Table S1), and the resulting targeting vectors were verified by sequencing (Fig. 1b).

In order to test the cleavage efficiency of each sgRNA, each of the four vectors was used in conjunction with pEGFP-N1 to individually co-transfect PFFs. Green fluorescent cells were directly observed 24 h after transfection. Genomic DNA was extracted from the PFFs after 72 h, amplified by PCR, and the products used to determine the mutation efficiency via the T7EI assay. The cleavage efficiencies of the four sgRNAs were 27.96, 44.89, 32.72 and 38.86%, respectively (Fig. 1c).

### CRISPR/Cas9-mediated MC3R gene targeting in pig fetal fibroblasts (PFFs)

Although the cleavage efficiencies of sgRNA2 and sgRNA4 were the highest of the four, sgRNA1 and sgRNA4 were chosen to generate the MC3R knockout (KO) cell line with a large-fragment deletion. The pX330-sgRNA1 and pX330-sgRNA4 targeting vectors were used together with the pL452 vector (expressing neomycin resistance) to co-transfect early-passage primary PFFs. A total of 72 colonies were isolated after G418 selection for 10 days. Genotype analysis of each colony was performed using TA-cloning and sequencing. The results indicated that 21 out of the 72 colonies harbored a homozygous/heterozygous biallelic mutation in the MC3R target gene region, including 11 MC3R^−/−^ and 10 MC3R^+/−^ colonies. The MC3R gene targeting efficiency of CRISPR/Cas9 therefore was 29.17%, with 15.28% biallelic mutations (Table 2). A total of 9 different large-fragment deletions (617–1005 bp) were identified by sequencing (Fig. 2a). A 999 bp deletion was the most frequent, occurring in 9 colonies, while the other mutations were present in only one colony each. Unexpectedly, an insertion of 2 nucleotides was observed in the strain with a 1005 bp deletion.

**Table 2:** MC3R targeting in PFFs using the CRISPR/Cas9 system

Monoallelic-KO	Biallelic-KO	Indel-positive
13.89%(10/72)	15.28%(11/72)	29.17% (21/72)

**Table 3:** Summary of the production of the MC3R gene knockout pig via CRISPR/Cas9

Transferred embryos	recipients	pregnancies	newborn pigs	mutants
1203	6	1	6	2

### Generation of MC3R knock-out pigs via somatic cell nuclear transfer (SCNT)

To generate MC3R knock-out piglets, biallelically modified colonies and wild-type control colonies were used simultaneously as donor cells for somatic cell nuclear transfer (SCNT). A total of 1203 reconstructed embryos were transferred into 6 surrogates. B-ultrasonic examination was performed on each recipient about 30 days after surgery, and 1 of the 6 gilts was found to be pregnant, corresponding to a pregnancy rate of 16.7% (Table 3). After 114 d, the recipient gave birth to 6 live piglets. One piglet was malformed and died 3 days after birth, while the others displayed no obvious defects. Genotyping results showed that the malformed piglet and one healthy male piglet were homozygous MC3R^−/−^ knockouts (Fig. 2b).

### Analysis of off-target events

To test whether off-target mutagenesis occurred in the MC3R KO piglet, potential off-target sites in the genome were screened using CRISPR Design software, revealing 5 candidate sequences. The PCR products of the potential off-target sites were analyzed by sequencing. Several nucleotide exchanges were detected, but no fragment deletions were observed (Fig. 2c). Insertions or deletions (indels) located around the sgRNA1 target site were considered to be NHEJ-mediated modifications.

### Body weight and adiposity analysis

**Fig. 3:**
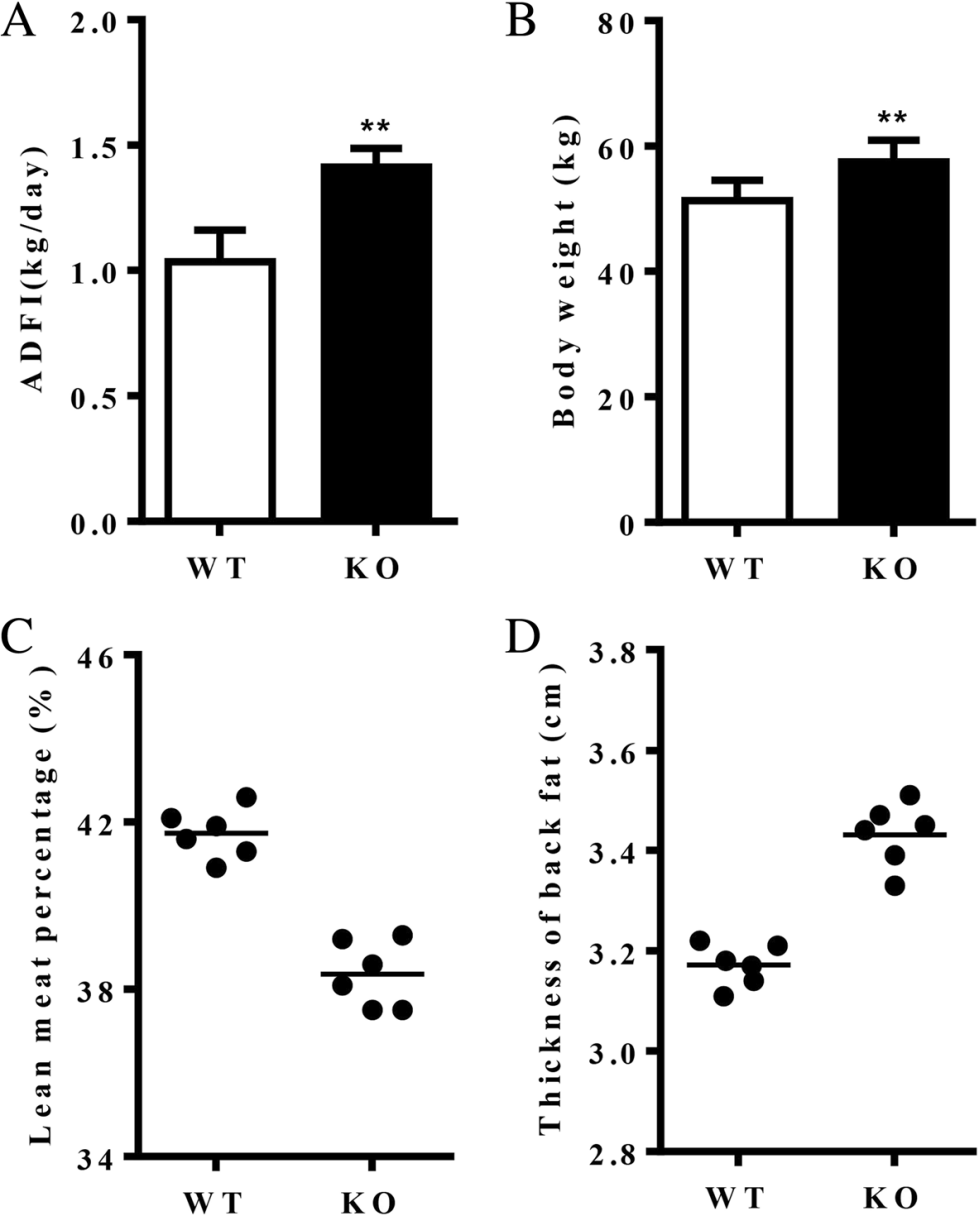
The effects of MC3R deficiency on the pigs’ growth. ADFI: Average daily feed intake, WT: wild type, KO: MC3R deficiency. Mean ± SD, *n* = 6. *t*-test, *P < 0.05, **P < 0.01. The panel labels (**a**-**d**) inside the image of Fig. 3 are “**a**-**d**: average daily feed intake, body weight, lean meat percentage and thickness of back fat analysis between MC3R KO and WT pigs”

To understand the effects of MC3R deficiency on pig growth, six male KO pigs were selected for food intake and adiposity analysis, and compared with their wild-type full siblings. According to the analysis of average daily feed intake and body weight (Fig. 3a and b), the KO pigs ate more and gained more weight than the wild-type pigs. The KO pigs had a lower lean meat percentage (Fig. 3c), which indicates that they deposited more fat than their wild-type full siblings. The thickness of back fat (Fig. 3d) was greater in the KO pigs, which confirmed that they deposited more fat than the wild-type pigs.

## Discussion

Obesity is the result of a complex interplay between genetic and environmental factors [24]. Polymorphisms in various genes controlling appetite and metabolism predispose to obesity when sufficient food energy is available. As of 2006, more than 41 sites in the human genome have been linked to the development of obesity when a favorable environment is present [25]. MC3R is one of these genes, and it is putatively involved in fat metabolism, but the research remains controversial. The evidence from knockout mice and rats supports the idea that MC3R is important for fat accumulation [12], but the phenotypes of MC3R KO mice and rats, such as the food intake, body weight and fat deposition, were not consistent [13]. Consequently, no conclusive opinion on the physiological function of MC3R is available to date.

It has been recognized that the metabolism of pigs is more similar to human metabolism than that of rodents [26]. In this study, a MC3R KO pig was generated, offering a new and possibly better model to study the gene’s function. Four sgRNAs were screened using CRISPR Design software, and the fragments obtained by annealing two complementary synthetic oligonucleotides were cloned into the pX330 vector with two *Bbs*I sites. There are many tools to choose target sites, including CRISPR Design [27] and E-Crispr [28], which offer scores for target sites. However, because CRISPR Design offers information on potential off-target sites, while E-Crispr does not, we used CRISPR Design to choose the target sites. The cleavage efficiencies of the four CRISPR/Cas9 target vectors in the PFFs were 27.96, 44.89, 32.76 and 38.86%, respectively. MC3R is a membrane receptor, and some of its residues may therefore play a role inside the cell membrane [29]. To avoid unexpected membrane effects of a truncated version of the protein, sgRNA1 and sgRNA4 target vectors were used to generate a large-fragment deletion in the MC3R genomic region. The plasmids pX330-sgRNA1 and pX330-sgRNA4 were used together with pL452 to co-transfect PFFs, resulting in a targeting efficiency of 29.17%, with 15.28% biallelic mutations, which was relatively low compared to other reports [30, 31]. This may be due to relatively inefficient enrichment of positive clones under G418 selection pressure, which indicates that our screening system may need to be optimized. Notwithstanding, colonies with large-fragment deletions ranging from 617 to 1005 bp were obtained. Compared to ZFNs and TALENs, CRISPR/Cas9 was demonstrated to be more efficient, enabling multiplex gene targeting [32]. Thus, the high targeting efficiency and ease of use of the CRISPR/Cas9 gene-editing tool will greatly facilitate the production of gene KO pig models in the future.

Using the KO cells, an MC3R knockout pig was generated by SCNT. Biallelically modified colonies were used along with wild-type control colonies as donor cells for nuclear transfer in order to obtain the MC3R KO and wild-type control pigs simultaneously. Because untreated PFFs usually exhibit higher SCNT efficiency than certain CRISPR/Cas9-treated PFFs [33], transferring pooled embryos derived from multiple cell lines of different clonability into a single surrogate could be a practical strategy to increase the chance to obtain genetically modified pigs. In this study, two types of MC3R KO colonies were used alongside wild-type PFFs as donor cells, and an MC3R KO pig was successfully generated using SCNT.

Off-target events are a major concern when using the CRISPR/Cas9 system, and many studies reported that high numbers of off-target mutations were induced in the genome [34]. To obtain information on off-target sites, ‘NGG’ and ‘NAG’ were chosen as the PAM sequences, and the whole genome of the pig was scanned. Five potential off-target sites were selected and tested by PCR-amplification and sequencing. We found that no off-target events occurred in the MC3R KO pig, and concluded that the vectors were relatively safe.

In order to understand the effects of MC3R deficiency on pig growth, average daily feed intake and body weight were analyzed. The lean meat percentage of the carcass and thickness of back fat, which have been widely used in pig carcass evaluation to indicate the fat ratio, were also analyzed. The results showed that both body weight and body fat of the KO pigs were greater than the corresponding values of wild-type pigs. MC3R mutations are known to cause obesity in humans [10], but rodent models with MC3R^−/−^ genotype failed to exhibit the same phenotype [12, 13]. Encouragingly, the MC3R-deficient pigs developed an obese phenotype, making them a potentially very valuable model for research on human obesity.

In summary, we successfully generated an MC3R KO pig strain using CRISPR/Cas9 technology in conjunction with SNCT. Since many questions remain about the relationship between MC3R and obesity, this new non-rodent model will be of great value for future research.

## Additional file

Additional file 1: **Figure S1** Alignment of pig MC3R sequences from GenBank and the CEM pig. **Table S1** Sequences of primers used in vector construction. **Table S2** Primer sequences used to amplify fragments surrounding sgRNA. **Table S3** Primer sequences for amplifying fragments surrounding OTSs (DOCX 33 kb).

## Data Availability

Not applicable (Data sharing not applicable to this article as all the data are already listed in this paper.)

## Abbreviations

CEM: Chinese experimental mini pigs
CRSPR/Cas: The clustered regularly interspaced short palindromic repeats associated protein system
KO: Knock-out
MC3R: Melanocortin 3 receptor
OTSs: Off-target sites
PFFs: Porcine fetal fibroblasts
SCNT: Somatic cell nuclear transfer
SNPs: Single nucleotide polymorphisms
TM: Transmembrane domain
UTR: Untranslated terminal region
