# Supplementary material for: Generation of an MC3R knock-out pig by CRSPR/Cas9 combined with somatic cell nuclear transfer (SCNT) technology
Source: Lipids Health Dis. 2019 May 28;18:122. doi: 10.1186/s12944-019-1073-9 (PMC6540458; doi:10.1186/s12944-019-1073-9)
Supplement: Supplementary file 1 — Figure S1 Alignment of pig MC3R sequences from GenBank and the CEM pig. Table S1 Sequences of primers used in vector construction. Table S2 Primer sequences used to amplify fragments surrounding sgRNA. Table S3 Primer sequences for amplifying fragments surrounding OTSs (DOCX 33 kb). [file 12944_2019_1073_MOESM1_ESM.docx]

**Supporting information**

Fig. S1 Alignment of pig MC3R sequences from GenBank and the CEM pig.

Sus (*Sus scrofa*) stands for the pig MC3R sequence (GenBank: EU091085), CEMP is the MC3R sequence of Chinese experimental mini Pig, Pr is the abbreviation of protein, red letters are the SNPs.

**Table S1. Sequences of primers used in vector construction.**

| **Name** | Sequence (5’→3’) |
| --- | --- |
| sgRNA**1F** | CACCGgcattcattgctcacggccg |
| sgRNA**1R** | AAACcggccgtgagcaatgaatgcC |
| sgRNA**2F** | CACCGggagaccatcatgatcgccg |
| sgRNA**2R** | AAACcggcgatcatgatggtctccC |
| sgRNA**3F** | CACCGggctccggaaggcgtagatc |
| sgRNA**3R** | AAACgatctacgccttccggagccC |
| sgRNA**4F** | CACCGcaggctaaggaccgctccgg |
| sgRNA**4R** | AAACccggagcggtccttagcctgC |

**Note:** Capital letters indicate sticky ends; lowercase letters indicate target sequences.

**Table S2. Primer sequences used to amplify fragments surrounding sgRNA**

| Name | Sequence (5’→3’) | Size | Amplified fragments (sgRNA) |
| --- | --- | --- | --- |
| XL1-F | GGAGAGGAAGGTAAGACAGGAGAG | 810bp | sgRNA1, sgRNA2 |
| XL1-R | TCTTGCTCTCGGAGTAGACGATG |  |  |
| XL2-F | GGGGGTCTTCATCTTCTGCTG | 757bp | sgRNA3, sgRNA4 |
| XL2-R | GAGGAGGGGCAAATAGCATCAC |  |  |

**Table S3. Primer sequences for amplifying fragments surrounding OTSs**

| Name | Sequence (5’→3’) | Size | sgRNA |
| --- | --- | --- | --- |
| OT1F | AAGGCTCTCTTCCGCAATGG | 662bp | sgRNA1 |
| OT1R | GGGTGGACGCAGCTATAACA |  |  |
| OT2F | TATTACCTCCCCTCCCCCAC | 531bp |  |
| OT2R | TGAGATCAAGCCAGCGCTAC |  |  |
| OT3F | GGAGCCGTGTGACTTATGGAA | 456bp |  |
| OT3R | GATGATAATGGACGCCAACGC |  |  |
| OT4F | AACTGGCACTAATCCTGCGG | 509bp | sgRNA4 |
| OT4R | ACTTTGAGGACTGGTTCCCG |  |  |
| OT5F | TCGTGTTTGTGTGCACTCCT | 598bp |  |
| OT5R | ACTTTCTCGAGTGGCGTCTG |  |  |
